# The Structure of Psychopathology on Reddit: Network Analysis of Mental Health Communities in Relation to the ICD Diagnostic System

**DOI:** 10.2196/80958

**Published:** 2026-01-30

**Authors:** Bojan Evkoski, Srebrenka Letina, Petra Kralj Novak

**Affiliations:** 1 Department of Network and Data Science Central European University Vienna Austria; 2 Department of Psychology University of Limerick Limerick Ireland; 3 Department of Knowledge Technologies Jožef Stefan Institute Ljubljana Slovenia

**Keywords:** psychopathology, disorder associations, online mental health support, Reddit, network analysis, ICD-10

## Abstract

**Background:**

Social media platforms such as Reddit have become important spaces where individuals articulate their distress, seek support, and explore alternative ways of understanding mental health outside traditional institutional frameworks. These environments provide an opportunity to examine mental health discourse at scale, offering perspectives that extend beyond traditional clinical and research settings.

**Objective:**

This study aims to examine the structure of mental health communities on Reddit by identifying patterns of association between mental disorders reflected in user activity and assessing how these relationships align with established diagnostic categories in the *ICD* (*International Classification of Diseases*).

**Methods:**

We manually curated 114 Reddit communities focused on specific mental health conditions from the 20,000 most active subreddits in 2022. Each community was labeled into 49 disorders and categorized under 9 *ICD* diagnostic categories within the group of mental and behavioral disorders, collectively known as the F codes. We constructed a disorder association network by identifying statistically significant user overlaps based on coposting across subreddit pairs using a bipartite configuration model, with Bonferroni-corrected significance (*P*<.001). We analyzed the connectivity of the network within and across diagnostic categories, examining inter- and intracategory links. Finally, we compared the structure of disorder associations inferred from Reddit with the *ICD* classification derived from diagnostic criteria using hierarchical clustering.

**Results:**

The inferred Reddit network of psychopathology revealed an interconnected structure (density=0.135), with all but 6 disorders forming a single giant component that spans across all 9 diagnostic categories. The most prominent disorders by number of users included hyperkinetic disorders (85,000), depressive episodes and recurrent depressive disorders (73,000), habit and impulse disorders (69,000), pervasive developmental disorders (52,000), and generalized anxiety disorder (44,000). In terms of connectivity, posttraumatic stress disorder (17/48 of all possible connections), obsessive-compulsive disorder (16/48), and depersonalization-derealization disorder (15/48) emerged as the most central in the network of positive disorder associations, while schizotypal disorder, avoidant personality disorder, and agoraphobia were the most central when accounting for the association strength. At the level of disorder categories, several disorders, such as bipolar disorder and premenstrual dysphoric disorder, displayed high intercategory associations but weak intracategory ties, indicating blurred diagnostic boundaries. The network of negative coposting associations revealed a divergence from the expectations of past research; for instance, addiction-related communities (eg, alcohol and opioids) were negatively associated with much of the broader mental health discourse. Finally, hierarchical comparisons showed moderate overlap between the Reddit network of disorder associations and the *ICD* network of diagnostic criteria, both in pairwise edge similarity (13% of edges present in both networks) and overall clustering (Adjusted Rand Index=0.295).

**Conclusions:**

Reddit-based mental health communities reveal a complementary structure of disorder associations shaped by lived experience, often diverging from formal diagnostic criteria and exhibiting patterns of association that do not align with established diagnostic boundaries.

## Introduction

Mental health problems have remained one of the main public health concerns, especially among younger populations [1-3]. This trend has unfolded amid rapid technological advancements and broader societal changes, including the lasting disruptions brought by the COVID-19 pandemic [4,5]. Yet, the pace of these societal and technological shifts has not been matched by corresponding adaptability in the broader health care system.

The challenge of adaptability is particularly evident in the diagnostic frameworks of psychopathology. Clinical taxonomies such as the *DSM* (*Diagnostic and Statistical Manual of Mental Disorders*) and the *ICD* (*International Classification of Diseases*) provide standardized frameworks for identifying, labeling, and treating psychological conditions. In these systems, disorders are defined and grouped into diagnostic categories based on symptom profiles, potential causal mechanisms or course of illness. These categories serve essential roles in practice: they guide diagnostic and treatment decisions, shape insurance coverage, structure research protocols, and enable communication across professionals. However, despite their utility, these frameworks and their diagnostic categories are built on standardized assumptions that inevitably generalize and oversimplify inherently subjective and dynamic experiences. Consequently, they have faced ongoing criticism regarding the ambiguity of their boundaries and their limited clarity, stability, and cultural relevance across diverse contexts [6-8]. Recent reform efforts, such as the Research Domain Criteria and the Hierarchical Taxonomy of Psychopathology, aim to address these limitations, but also underscore the complexity and ongoing contestation surrounding psychiatric classification in the pursuit of a more fluid, flexible, and context-sensitive approach to understanding mental illness [9-11]. However, such efforts cannot be fully undertaken in isolation from the shifting social and technological environments that shape how symptoms are expressed, experienced, and interpreted.

Over the past 2 decades, digital technologies have transformed nearly every facet of daily life, including how individuals relate to their own mental health. Mobile connectivity and online social media play central roles in how people articulate their experiences, shape their identities, and seek support [12,13]. Platforms such as Reddit have emerged as key infrastructures for navigating psychological problems, particularly for individuals who may not have access to, or trust in, formal health systems [14]. The appeal of these spaces is amplified by the limitations of traditional care: clinical services often prioritize severe cases, socioeconomic barriers restrict access, and not all individuals are equally willing or able to seek professional help. Even when accessed, brief and episodic consultations may leave little room to capture the full scope of ongoing psychological struggles [15-17]. By contrast, the anonymity and decentralization of digital health communities enable frank discussions of stigmatized experiences, which allows users to articulate symptoms and explore possible explanations. In doing so, these platforms contribute to a contemporary, living discourse around mental health.

Because users can engage freely, anonymously, and repeatedly over time, digital health communities provide a naturally occurring data source for examining both the expression of diverse conditions and their interrelations [18,19]. While research on online platforms has typically emphasized diagnostic tools or peer support within isolated communities or narrow disorder sets [20-22], recent cross-community studies have started studying online mental health communities through comparative and cross-community perspectives. For example, Morini et al [23] analyzed the content of 67 mental health communities on Reddit and showed that support-seeking and venting are dominant posting intents, and that community feedback shapes subsequent participation. In parallel, Jin and Zhu [24] constructed a multimorbidity network linking diabetes communities to 88 other disease subreddits, revealing connections to mental health and weight-management forums and showing that discussion of physical illness can extend into mental health–focused communities. Such cross-community studies have the potential to complement traditional research and open the way for examining large-scale, system-level patterns of psychopathology and the diagnostic frameworks that represent them.

Empirical research on diagnostic frameworks and relationships between disorders has largely relied on 2 sources: surveys and clinical registers. Surveys capture subsets of symptoms in specific populations and depend on self-report, which is difficult to validate at scale [25-27]. On the other hand, while clinical registers are based on verified diagnoses, they typically contain biases based on severity, health care access and even diagnostic conventions [28,29]. As such, both perspectives offer important yet partial views on the structure of psychopathology. We position digital mental health communities, specifically Reddit, as a complementary source that provides behavioral signals of perceived relatedness among mental disorders. These data are not confined to small samples or predefined diagnostic categories, and they are not restricted to clinically severe cases. Instead, they capture large-scale, naturally occurring expressions of personal experience that cannot replace survey or register approaches, yet can broaden the empirical basis for psychopathology research and enable triangulation across complementary sources.

We study the structure of psychopathology on Reddit by analyzing users’ coposting activity in condition-specific mental health communities. The dataset comprises 114 subreddits, each centered on a distinct disorder, collectively covering more than half a million users and over 1.5 million posts. By tracing associations across disorders through shared patterns of coposting, we adopt a data-driven network perspective on how conditions are interconnected in contemporary contexts. This perspective draws on tools from network analysis that have grown influential in mental health research, particularly network psychometrics, where disorders are modeled as systems of interacting symptoms rather than latent disease categories [30-32]. Whereas network psychometrics emphasizes within-disorder architecture, we shift the focus to relationships between disorders as reflected in cross-community engagement. In doing so, we complement symptom-level approaches of psychopathology with a view of how people navigate multiple diagnostic ideas at once, negotiating meaning and seeking support across disorder boundaries.

Our main objective is to infer a significance-based network of associations between mental health conditions as expressed through online engagement. We identify pairs that co-occur more or less often than expected, as well as mental health conditions that act as high-degree bridges across diagnostic categories. We highlight clusters of co-occurring disorders and examine strong cross-category connections that may signal transdiagnostic roles not anticipated by existing taxonomies. Finally, we compare the Reddit-derived structure with the hierarchy encoded in the *ICD-10* (*International Statistical Classification of Diseases, 10th Revision*) diagnostic criteria, outlining conceptual and structural differences that contribute to the broader discussion on psychiatric nosology. In this way, Reddit functions as both a site of peer support and a window into how people collectively make sense of mental health beyond formal clinical narratives.

## Methods

### Study Design

The study design centers on the structure of user coposting across disorder-specific subreddits as a behavioral proxy for latent relationships between mental health disorders. We treat statistically significant user overlap between subreddit pairs as evidence that seeking advice for one disorder is associated with seeking advice for another, indicating that the 2 disorders are conceptually proximate. Such proximity may rise from several mechanisms, such as comorbidity, where users experience or suspect multiple concurrent disorders; diagnostic progression, where users transition between diagnoses over time; and misdiagnosis, where users reconsider or question a diagnosis, whether self-identified or clinically provided. Although some overlap could reflect general cross-community engagement, previous research shows that most mental health content on Reddit centers on first-person struggles rather than general discussion [14,23]. Our own validation supports this pattern: more than two-thirds of posts contain more self-referential pronouns (“I,” “me,” and “myself”) than references to others (refer to Multimedia Appendix 1), consistent with help-seeking grounded in personal experience. To limit residual unrelated activity, we infer edges only when observed user overlap exceeds expectations under a conservative bipartite configuration model with stringent multiple-comparison control (refer to the “Network Inference” subheading below). As a final major design choice, we decided to focus on activity through posts rather than comments. Posts in disorder-specific communities are the primary venue for sharing experiences and seeking help, whereas comments are more reactive, often offering advice or feedback. While comments provide valuable perspectives on peer interaction and information diffusion, their scale and heterogeneity risk diluting the signal of the help-seeking behavior we aim to capture. Together, these design choices justify interpreting coposting as a cautious but credible signal of perceived relatedness between disorders without interpreting activity as evidence of diagnosis or medical history.

### Data

We collected Reddit data through the Pushshift application programming interface by looking into the 20,000 biggest subreddits [33,34]. From this corpus, we manually curated a list of subreddits whose primary focus is to provide information, support, and shared lived experience related to specific mental health conditions. Each candidate subreddit was reviewed individually, considering both its description and posted content, and was included only if those corresponded to a distinct disorder as described in the *ICD-10* (2019 release) [35].

Each included subreddit was annotated according to its corresponding *ICD-10* diagnostic code at level 4 granularity (eg, F48.1, depersonalization-derealization syndrome). To study the complete hierarchy of psychopathology, we further annotated each community to higher-level categories of the *ICD* taxonomy, including level 3 codes (eg, F48, other neurotic disorders) and level 2 codes (eg, F4, neurotic, stress-related, and somatoform disorders). For completeness and future interoperability, we also provided mappings to the equivalent codes in the *ICD-11* (*International Classification of Diseases, 11th Revision*), a taxonomy that is yet to be used in practice. In total, 114 condition-specific mental health subreddits were identified and classified into 49 unique *ICD-10* disorders, covering 9 level 2 diagnostic categories of mental and behavioral disorders (from F0 to F9). A full list of annotated subreddits and their hierarchical coding is provided in Multimedia Appendix 2 and is publicly available for reuse, along with a separate table listing all disorders and their corresponding *ICD-10* codes.

We restricted our analyses to posts from 2022 to ensure temporal consistency and avoid confounding effects from major platform-level disruptions. This includes discontinuities associated with the COVID-19 pandemic and subsequent policy or moderation shifts, as well as more recent artifacts linked to the rise of generative artificial intelligence [36,37]. The resulting dataset comprised 1,513,016 posts authored by 545,330 unique users across all included mental health subreddits. On average, each user contributed 2.77 posts, with 96,742 (17.74%) users posting in more than 1 mental health subreddit, which forms the foundation for our analysis of disorder co-occurrence (refer to Multimedia Appendix 3 regarding the distribution of posts across disorder categories and subreddits). To enhance data quality, we excluded accounts indicative of automated or spam-like activity. Specifically, the dataset excluded users who posted more than 365 times in the study year (corresponding to a rate of more than 1 post per day), as well as known automated accounts listed in the publicly available bot directory BotRank [38]. Ultimately, the data collection procedure resulted in a comprehensive representation of Reddit’s mental health discourse by systematically covering the major condition-specific communities active on the platform.

### Network Inference

#### Overview

The main objective of this work is to infer relationships between mental health conditions as they emerge from patterns of shared user participation across the studied disorder-related subreddits. To this end, we constructed a weighted network in which each node represents 1 of the 49 classified mental health disorders, defined at the level 4 granularity of *ICD-10* codes, and edges capture statistically significant user coposting overlaps between the sets of subreddits corresponding to each disorder pair. The construction involved 4 key steps: computing user overlap between disorder pairs, estimating a null model for expected coposting, determining statistical significance, and assigning edge weights based on deviation from null expectations.

#### Disorder Association Metric

For each pair of disorders, we measured the strength of coposting association by calculating the overlap coefficient between the sets of users who contributed to subreddits of each corresponding disorder. This coefficient captures the size of the user intersection normalized by the smaller of the 2 user sets:


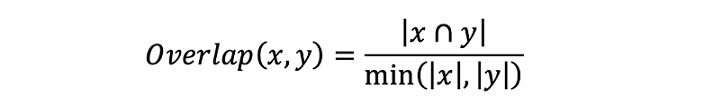


where *x* and *y* denote the sets of unique users who posted in the subreddits associated with each disorder.

The overlap coefficient is particularly suited for capturing asymmetric coengagement. By measuring the proportion of shared users relative to the smaller community, we can address cases where participation in one subreddit could be almost entirely embedded within another. This property aligns well with the hierarchical and overlapping conceptualization of many mental health conditions, where narrower or less prevalent disorders often exist within the broader spectrum of more common ones.

#### Null Model and Statistical Testing

To assess the significance of observed user overlaps, we used a binary bipartite configuration model as the null model. The user-disorder bipartite network was constructed with one set of nodes representing users and the other representing mental health disorders, where an edge denotes a post by a user in a subreddit labeled with a specific disorder. To generate the null distribution, we randomly rewired the bipartite network 10,000 times while preserving the degree distributions of both users and disorders and preventing multiedge cases. Each rewired network underwent several edge swaps equal to 10× the total number of edges, ensuring sufficient randomization while maintaining the original participation heterogeneity. This approach follows established practices in bipartite network analysis, where degree-preserving randomization is commonly used to construct realistic null models for statistical inference [39,40].

For each disorder pair, we computed the distribution of overlap coefficients across the 10,000 null replicates and evaluated the statistical deviation of the observed overlap using a standard z score. To correct for multiple comparisons across all possible disorder pairs (n × [n – 1] / 2), we applied a Bonferroni-corrected significance threshold of *P*<.001 [41]. Based on this, each disorder pair falls into one of 3 categories:

- Positive association: observed overlap is significantly higher than expected.
- Negative association: observed overlap is significantly lower than expected.
- No evidence for association: observed overlap does not differ significantly from null expectations.

#### Edge Weights and Network Representation

For each statistically significant association, we assigned a weight equal to the difference between the observed and the mean expected overlap coefficient. This measure reflects the magnitude of deviation from the null, with higher values indicating stronger-than-expected co-occurring between disorders. Theoretically, the weights range from 0 (no deviation) to 1, with larger values signaling greater empirical association strength relative to what would be expected by chance under the null model.

The resulting structure was represented as two undirected weighted networks of positive and negative disorder associations. This network representation enabled intuitive interpretation of pairwise association patterns, capturing the relational landscape of psychopathology. An interactive online version of the network visualization was also developed to facilitate further exploration (refer to Multimedia Appendices 4 and 5) [42-44].

#### Node-Level Metrics

To characterize the centrality of disorders within the inferred network, we computed 2 measures of connectedness:

- Unweighted degree: the total number of significant associations (edges) for a given disorder. This captures how broadly a condition is connected across the mental health landscape.
- Weighted degree: the sum of edge weights for all associations of a disorder. This emphasizes the cumulative strength of its connections, highlighting conditions that might not have many links, yet maintain particularly strong associations.

Across analyses, we used both unweighted and weighted edges depending on the methodological requirements of each approach. Comprehensive data for both unweighted and weighted node degrees of the Reddit network are provided in Multimedia Appendix 2.

### ICD-10 Network of Diagnostic Criteria

As a reference point for the Reddit-derived association network, we constructed a network of mental disorders based on formal diagnostic criteria, referred to as the *ICD-10* diagnostic criteria network. This network was built using data curated by Tio et al [45], who systematically extracted diagnostic symptoms for each *ICD-10* code falling into Chapter F: mental and behavioral disorders. This dataset provides a standardized operationalization of disorder-level symptom profiles for *ICD-10* codes that remain in clinical use [46]. It offers a unique opportunity to analyze relationships between disorders grounded in clinical definitions, which are otherwise difficult to access in a machine-readable or systematically coded form. As such, we used this network for comparing the *ICD-10* diagnostic structure with the association patterns inferred from the coposting activity on Reddit.

In the *ICD-10* diagnostic criteria network, each node (disorder) was represented as a set of diagnostic criteria, and the edge weight (strength of connection) between 2 disorders was calculated as the overlap coefficient between their diagnostic criteria sets. This formulation mirrors our approach to the Reddit network, enabling a consistent comparison across both systems. To ensure a valid basis for comparison, we restricted this network to include only those disorders for which a corresponding Reddit community had been identified in our manual curation.

### Hierarchical Clustering of Association Networks

We used hierarchical clustering to study the potential modular and hierarchical structure of the association network derived from Reddit, as well as that of the *ICD-10* diagnostic criteria network. This approach, which has previously been used to uncover grouping patterns in association networks [47-49], allowed us to infer latent groupings of mental health conditions based on observed similarity patterns. Importantly, it enabled a system-level comparison of the 2 networks, moving beyond pairwise overlap to examine how broader patterns of connectivity and disorder organization differ between Reddit discourse and the formal *ICD-10* diagnostic structure, akin to comparing hierarchical network communities.

We applied standard agglomerative clustering with average linkage, where 2 clusters were merged based on the average distance between all pairs of disorders across the 2 clusters [50]. Since the weighted edges in both networks represent similarity (rather than distance), we defined the distance between any 2 disorders *x* and *y* as:

*d*(*x*,*y*) = *max*(*e*(*x*,*y*)) – *e*(*x*,*y*)

Where *e*(*x*,*y*) is the observed similarity (edge weight) and *max*(*e*) ensures that distances are positive and properly scaled for clustering.

To assess the extent to which each network exhibits clustered structure, we first computed the weighted modularity, a standard quality function that quantifies how much edge weight lies within clusters compared to between clusters, relative to the expectation under a weighted degree-preserving null model [51]. This allowed us to evaluate and compare the overall modular organization of the Reddit network and the *ICD-10* diagnostic criteria network.

For a clustering π (nodes grouped into clusters), the weighted modularity is defined as:


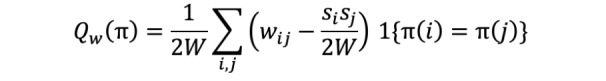


Where *w*_i_,_j_ is the observed edge weight, *s_i_*=∑*_j_w_i_*_,_*_j_* is the weighted degree of node *i*, *2W*=∑*_ij_w_ij_* is the total edge weight, and 1{⋅} equals 1 when *i* and *j* are assigned to the same cluster. Intuitively, higher weighted modularity values indicate a clearer community structure. *Q_w_* is large when within-cluster connections carry more weight than expected under a null model preserving node strengths.

Since raw modularity depends on network density and degree or strength heterogeneity, we estimated the normalized modularity for each network under a degree-sequence–preserving null model to enable a fair comparison between the Reddit network and the *ICD-10* network of diagnostic criteria. For each network, we first generated an ensemble of randomized topologies by repeatedly swapping edge pairs and their associated weights, thereby preserving the overall degree sequence and weight distribution while randomizing the network. Then, for each randomized graph *G'*, we evaluated *Q_w_*(*G'*,*π*(*τ*^⋆^)) on the same partition *π*(*τ*^⋆^) obtained from the observed network; this isolates how expected the observed within-cluster concentration of weight is, given the degree and weight distribution.

We then calculated the normalized modularity, defined as the difference between the observed modularity and the expected value under the null model:

*ΔQ_w_*=*Q_w_*(*G*,*π*(*τ*^⋆^))–*E*[*Q_w_*(*G'*,*π*(*τ*^⋆^))]

where the latter was estimated from 1000 randomized realizations. Finally, for each dendrogram height produced through hierarchical clustering, we evaluated the normalized weighted modularity and compared the Reddit network with the *ICD-10* network of diagnostic criteria, focusing on the clustering results corresponding to the dendrogram cuts with the highest normalized weighted modularity in each network. In other words, the final clustering of a network is obtained as the cut τ⋆ that maximizes *ΔQ_w_* across all admissible cuts:


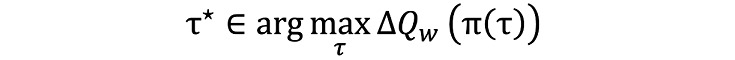


To evaluate the similarity between the final clusters of the 2 networks, we used 2 standard comparison indices: the Adjusted Rand Index (ARI) and the normalized mutual information (NMI) [52]. ARI measures the agreement between 2 clustering results by quantifying how often pairs of nodes are grouped or separated in the same way, adjusted for chance. NMI captures the amount of shared information between the clusters of the networks and is less sensitive to differences in the number or size of clusters. Both metrics range from 0 (no agreement beyond chance) to 1 (perfect correspondence).

### Ethical Considerations

The subreddits analyzed in this study are publicly accessible and do not require login credentials. Posts are shared under pseudonymous accounts, and all usernames were further pseudoanonymized before analysis to protect privacy. Given the sensitive and personal nature of the disclosures that may appear in these vulnerable communities, we applied strict privacy safeguards and limited all reporting to aggregated results, without focusing on individual cases and basing our analysis only on coposting rather than the content of the posts themselves. The study was purely observational, involving no interaction or intervention with users. We did not attempt to contact individuals, and no analyses were conducted that could enable reidentification of participants. Our approach followed widely recognized ethical frameworks for internet-mediated research [53-56]. In particular, we adhered to the Tri-Council Policy Statement: Ethical Conduct for Research Involving Humans (TCPS 2), which emphasizes proportional safeguards for minimizing risks when analyzing data from public platforms where individuals can reasonably expect to be observed without explicit consent [57]. This research was also reviewed and approved by the Department of Network and Data Science Ethical Research Committee at the Central European University (reference no 2024-2025/16/ RD/DNDS).

## Results

### The Reddit Network of Psychopathology

We constructed a data-driven network of mental health disorder associations based on user coposting behavior across 114 Reddit communities. Each node in the network corresponds to a disorder-level *ICD-10* code (Chapter F), and edges represent statistically significant coposting links, derived from observed versus expected user overlap.

The derived Reddit network of positive associations consists of 49 nodes and 159 edges, with a density of 0.135. Despite this relative sparsity, the network forms a single giant component encompassing 43 of the 49 disorders, exemplifying the interconnectedness of mental health conditions. Only 3 disorders remained isolated without any associations, indicating a lack of shared user engagement with other conditions. These isolated disorders are F00-F03 (dementia), F63.0 (gambling addiction), and F98.5 (stuttering).

**Figure 1:**
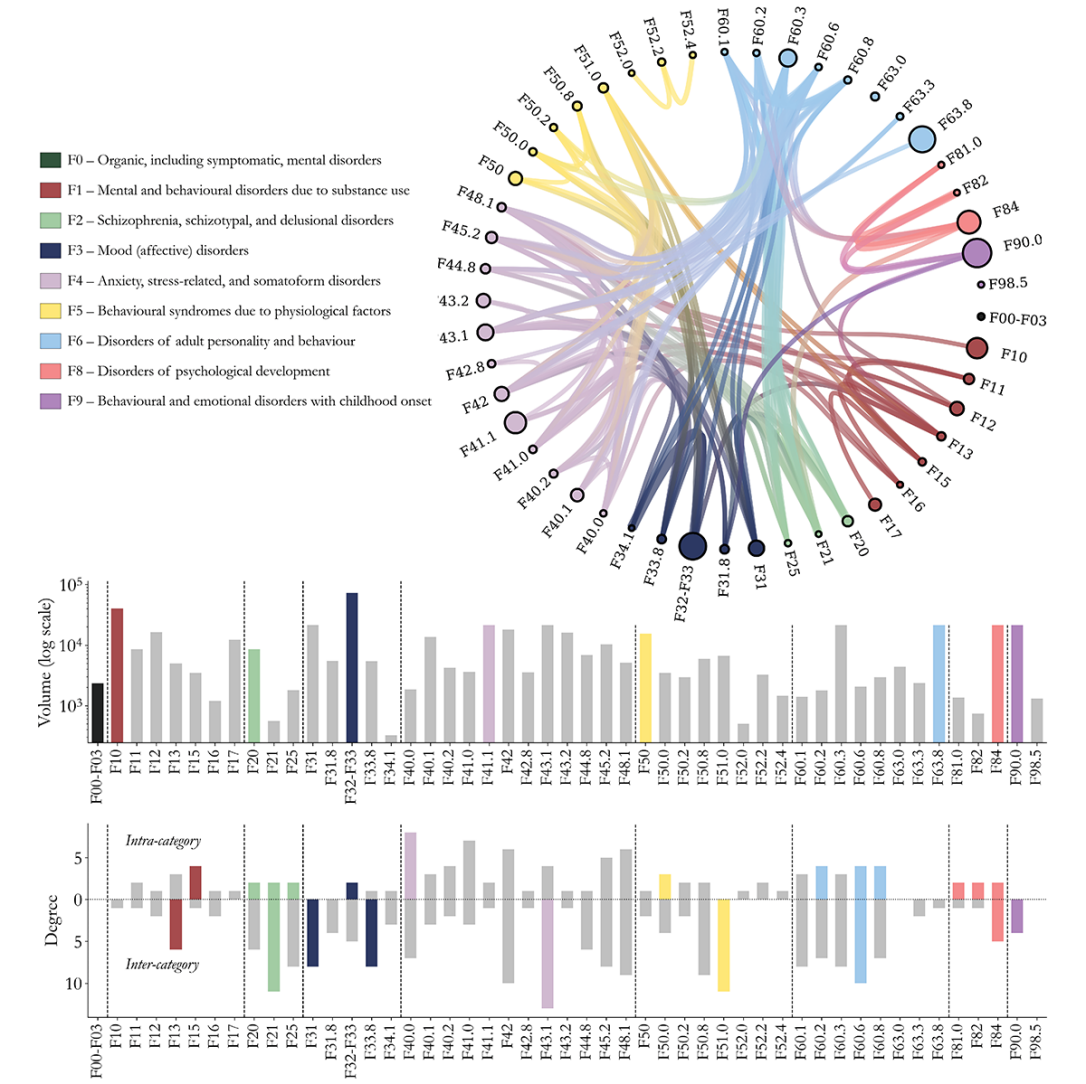
Network of positive associations among disorders derived from Reddit coposting activity in 2022, covering 114 condition-specific communities (subreddits) mapped to ICD-10 (International Classification of Diseases, 10th Revision) Chapter F (Mental and Behavioral Disorders). The network includes 49 disorder nodes linked by 159 edges. Top: a circular (radial) network visualization. Each node represents a distinct ICD-10 disorder, grouped and color-coded by higher-level diagnostic category (F1-F9). Node size indicates the number of unique users who posted at least once in the corresponding subreddits. Edges connect pairs of disorders that share a statistically significant overlap in user activity, estimated with a bipartite configuration null model and corrected for multiple testing (Bonferroni-adjusted, *P*<.001). Edge density and cross-category links illustrate the extent of interconnectedness across diagnostic boundaries. Bottom: disorder-level statistics. The center panel shows the number of users associated with each disorder. The bottom panel shows the degree of each disorder, defined as the number of significant co-occurring associations, broken down into intracategory associations (links within the same ICD-10 level-2 group) and intercategory associations (links connecting disorders across different ICD-10 groups; eg, between F1 and F5). Colored bars highlight the maximum values within each diagnostic category. Together, these panels summarize both the structure of the Reddit-based disorder network and the relative prominence of individual disorders by participation size and connectivity.

Figure 1 (top panel) shows a circular layout of the Reddit network. Node size corresponds to the total number of users active in that disorder’s subreddits, and node color denotes its *ICD-10* diagnostic category. The disorders with the highest number of users include F10 (alcohol addiction), F32-F33 (depressive episodes and recurrent depression), F63.8 (other habit and impulse disorders, including excessive masturbation and pornography addiction), F84 (pervasive developmental disorders in *ICD-10*; termed autism spectrum disorder in *ICD-11*), and F90.0 (hyperkinetic disorders in *ICD-10*; termed attention-deficit/hyperactivity disorder [ADHD] in *ICD-11*). While the distribution of user activity is heterogeneous, we observed no significant association between the number of users engaging with a given disorder and the number of connections in the network. This lack of association between volume and connectivity supports the robustness of the inference method, suggesting that network centrality reflects patterns of coposting that cannot be explained simply by subreddit size (refer to Multimedia Appendix 6 for correlation results between degree and volume).

Figure 1 (bottom panel) shows the degree distribution of nodes in the network, revealing substantial heterogeneity in how strongly different disorders are connected, ranging from highly linked hubs to sparsely connected nodes (refer to Multimedia Appendix 2 for results on weighted degrees). The most connected disorders include F43.1 (posttraumatic stress disorder [PTSD]), F42 (obsessive‑compulsive disorder), F40.0 (agoraphobia), F48.1 (depersonalization‑derealization syndrome), and F60.6 (avoidant personality disorder). These disorders emerge as transdiagnostic hubs, a concept central to psychiatric nosology, with associations spanning a wide range of other conditions. In contrast, disorders with minimal connectivity, such as F10 (alcohol addiction), F17 (tobacco addiction), and F52.0 (lack or loss of sexual desire), may reflect more segmented or marginalized user groups, narrower community focus, or greater diagnostic specificity, similar to the 3 isolated disorders identified before in this subsection.

The network of positive associations reveals a strong presence of cross‑category links, with 106 of 159 edges connecting disorders classified in different *ICD‑10* diagnostic categories. Notably, some disorders show markedly higher intercategory associations than intracategory ones. For example, F43.1 (PTSD), F51.0 (nonorganic insomnia), and F33.8 (used here to denote premenstrual dysphoric disorder) exhibit the largest discrepancies, with strong cross-category ties but weak integration within their respective *ICD-10* groups. This pattern also extends to entire higher-level diagnostic categories: all conditions in F2 (schizophrenia, schizotypal, and delusional disorders) and F3 (mood and affective disorders) display more intercategory than intracategory connections, highlighting particularly fluid boundaries for these diagnostic categories.

### Negative Associations

While our primary focus was on positive associations, indicative of shared user bases and potential comorbidity patterns, we also identified a set of negative associations, where user coposting occurred less frequently than expected under the null model. However, due to methodological limitations, such results should be interpreted at the level of individual edges only, not aggregated across nodes. Specifically, the inference strategy using a null model and the overlap coefficient to measure association becomes increasingly insensitive to low coposting rates in smaller or less active subreddits, introducing a lower-bound bias that distorts node-level summaries of negative associations (refer to Multimedia Appendix 7).

Despite this limitation, a consistent pattern emerges. Negative associations were most commonly observed between impulse-related disorders (eg, F10 for alcohol use, F11 for opioid use, and F63.8 for other habit and impulse disorders) and other mental health categories. These patterns may reflect distinct user populations, stigma-driven disengagement, or divergent framings of psychological distress and its management. The presence of such negative ties underscores that disorder communities on Reddit are not only interconnected but also socially and discursively fragmented in ways that do not always align with theoretical similarity and comorbidity reported in the literature. A notable example is F11 (opioid use), which showed negative associations with 7 of 12 nodes in the F4 category (neurotic, stress-related, and somatoform disorders), despite previous research suggesting strong links between anxiety and opioid use [58,59]. A full list of negative edges and their weights is provided in Multimedia Appendix 2. Considering the limitations of our analysis on negative associations, these findings warrant further investigation using alternative methodological approaches better suited to capture negative associations in smaller samples.

### The Hierarchical Structure of Psychopathology: Comparison With ICD-10 Diagnostic Criteria

To better understand the structural logic underlying user-inferred relationships between mental health conditions, we examined the emergent hierarchical organization of the Reddit coposting network and compared it with a clinically derived alternative based on diagnostic criteria overlap. While pairwise associations are informative, a hierarchical view enables assessment of whether larger clusters of disorders emerge in user behavior and how these clusters compare with the taxonomy in the *ICD-10* system.

**Figure 2:**
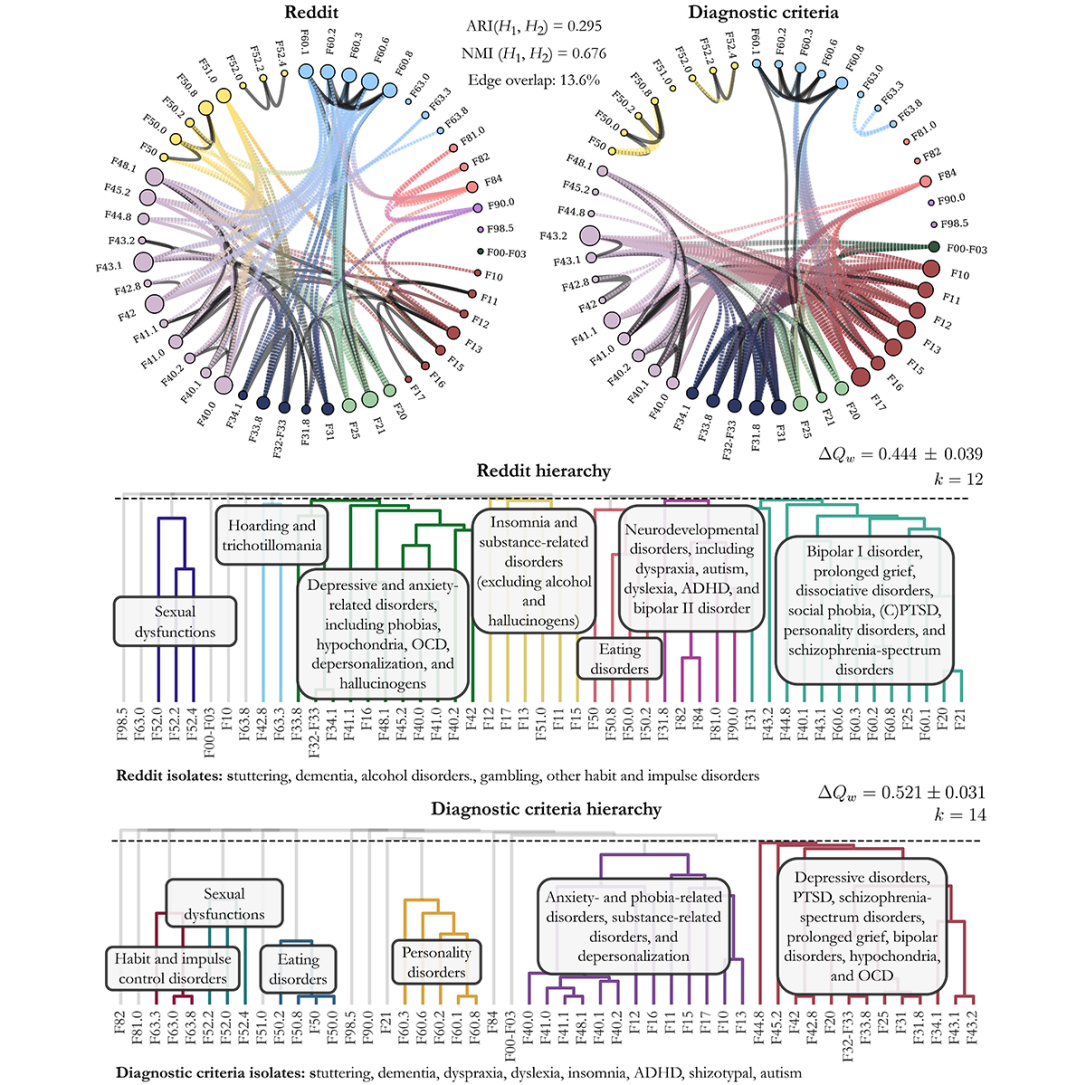
Comparison between 2 networks of mental health disorder relationships: 1 derived from Reddit user activity and 1 constructed from clinical diagnostic definitions, illustrating both overlaps and divergences between lived-experience associations emerging from online peer support and the formal structures encoded in psychiatric nosology. Top panels: circular network visualizations. The network on the left is based on coposting behavior across 128 condition-specific Reddit communities (subreddits) active in 2022, mapped to International Classification of Diseases, 10th Revision (ICD-10) Chapter F (Mental and Behavioral Disorders). Two disorders are linked if users post in both corresponding subreddits more often than expected by chance. The network on the right is the diagnostic criteria network, based on a curated list of disorders and diagnostic criteria of ICD-10, where disorders are linked if they share at least 1 formal diagnostic feature. In both networks, node size reflects connectivity (degree), and color indicates the ICD-10 diagnostic category (F0-F9). Black edges highlight associations that are common to both networks, while colored edges are unique to either Reddit (orange) or the diagnostic criteria (blue). Bottom panels: hierarchical clustering of disorders in both networks. Agglomerative clustering with average linkage was applied to identify higher-level clusters, with the cut-off chosen to maximize normalized weighted modularity (a measure of how well the network separates into cohesive clusters beyond chance). The resulting normalized weighted modularity values were ∆Q_w=0.420 for Reddit and ∆Q_w=0.521 for the diagnostic criteria network, showing that the Reddit network is more interconnected across diagnostic categories compared with the more modular diagnostic criteria–based network. Cluster labels are simplified, human-readable summaries of underlying ICD-10 codes. ADHD: attention-deficit/hyperactivity disorder; ARI: Adjusted Rand Index; NMI: normalized mutual information; OCD: obsessive-compulsive disorder; PTSD: posttraumatic stress disorder.

Figure 2 (top) presents both the Reddit and diagnostic criteria–based networks using circular layouts. Both networks contain the same set of 49 *ICD‑10* codes but differ in how connections are formed: the Reddit network uses statistically significant coposting links based on user overlap, while the diagnostic criteria network connects disorders based on shared clinical features, as curated by Tio et al [45]. Edges in both networks are weighted by the overlap coefficient, which quantifies the proportion of shared elements between 2 sets relative to the smaller set.

We applied agglomerative clustering with average linkage to both networks and identified the optimal clustering by cutting the dendrogram at the level of maximum normalized weighted modularity (∆*Q_max_*). The resulting modularity scores were 0.444 (12 clusters) for Reddit and 0.521 (14 clusters) for the diagnostic criteria network (Figure 2, bottom). This finding suggests that the Reddit network is less modular, reflecting less distinct clusters and more overlapping patterns of association compared with the more compartmentalized structure based on symptom overlap.

Overall, the comparison of the 2 hierarchies showed partial alignment between Reddit‑based coposting behaviors and the formal diagnostic structure, with areas of both convergence and divergence. Only 13% of links were shared between the 2 networks, highlighting a substantial dissimilarity in their underlying associations. This limited edge overlap suggests that user-inferred connections diverge notably from those based on diagnostic criteria. However, when comparing their hierarchical structure, we observe more alignment. The ARI of 0.295 and the NMI of 0.676 indicate moderate similarity between the clusters of the 2 networks, pointing to partial similarity in how disorders are categorized despite the underlying differences in pairwise associations.

The Reddit network also presents a more convoluted structure, with only 5 disorders remaining unclustered at the modularity-optimal cut: F98.5 (stuttering), F00-F03 (dementia), F10 (alcohol addiction), F63.0 (gambling addiction), and F63.8 (other habit and impulse disorders). In contrast, the diagnostic criteria network leaves 8 disorders unclustered, with only 2 repeated from the Reddit network (stuttering and dementia). Importantly, some of the conditions that remain unclustered in the *ICD-10*–based network occupy more central positions in the hierarchy of the Reddit network. These include F21 (schizotypal disorder), F43.1 (PTSD), F44.81 (dissociative identity disorder), F84 (autism spectrum), and F90.0 (ADHD). Although still considered peripheral in traditional diagnostic systems, several of these conditions have received growing attention in recent years, either for their proposed transdiagnostic relevance [60,61] or for their apparent connection to broader technological and social changes, including increased screen exposure and digital media use [62-64]. Their presence in Reddit-based clusters may indicate that users organize mental health experiences in ways that diverge from formal diagnostic structures, shaped instead by evolving discussions around neurodivergence, trauma, and identity.

Some of the clusters in the Reddit hierarchy echo broader dimensional models of psychopathology. For example, F32-F33 (depressive disorders), F41.0-F41.1 (anxiety disorders), and F42 (obsessive-compulsive disorder) form a prominent cluster, an intersection that is absent from the *ICD-10* diagnostic structure but aligns with internalizing spectra described in dimensional models [10]. Another Reddit-derived cluster connects psychotic conditions such as F20 (schizophrenia) and F25 (schizoaffective disorder) with trauma-related disorders such as F43.1 (PTSD) and F44.81 (dissociative identity disorder), as well as personality disorders such as F60.3 (emotionally unstable personality disorder). This forms a cluster of thought disorders situated at the boundary between conditions typically conceptualized as internalizing or externalizing.

## Discussion

### Principal Findings and Relevance to Psychopathology Research

To examine how anonymous users collectively make sense of mental health problems and how this organization relates to clinical diagnostic manuals, we analyzed activity in 114 disorder-focused Reddit communities involving 545,000 users and more than 1.5M posts. We inferred a significance-based network of associations among 49 disorders spanning 9 *ICD-10* mental and behavioral disorder categories (F0-F9), derived from patterns of coposting across communities. We then compared this Reddit-based structure with a network constructed from overlaps in diagnostic criteria defined in the *ICD-10*. The Reddit network revealed a highly interconnected organization that crossed traditional diagnostic boundaries. Several disorders occupied central positions, acting as hubs that linked otherwise distinct diagnostic categories, while others, particularly substance- and behavior-related addictions, appeared less integrated into the broader mental health discourse. Comparisons of the hierarchical structure showed only partial correspondence between the Reddit network and *ICD-10*, indicating that patterns of association emerging from lived experience differ in systematic ways from those encoded in formal taxonomies. Viewed in this large-scale context, digital mental health communities do not simply mirror existing diagnostic structures. Instead, they organize mental distress through shared experience and collective interpretation, producing a socially situated view of how disorders relate to one another. This perspective extends beyond what can be captured through surveys or clinical registers alone and can help refine how comorbidity patterns and disorder boundaries are understood outside formal clinical settings.

In more detail, the statistical inference of the Reddit network revealed 159 associations between the 49 studied disorders. Despite its moderate density (0.135), the network formed a large, connected component that encompassed 43 of the 49 conditions, indicating that most conditions were linked to one another through chains of significant associations that cut across traditional diagnostic categories. Complementary patterns have been reported in symptom-level network research, where psychopathology appears as a highly interconnected system rather than clearly separated clusters [65].

Further looking into the structure of the Reddit network, several disorders emerged as central transdiagnostic hubs, including F43.1 (PTSD), F42 (obsessive‑compulsive disorder), F40.0 (agoraphobia), F48.1 (depersonalization‑derealization syndrome), and F60.6 (avoidant personality disorder). Their high centrality reflects symptoms that cut across diagnostic boundaries, such as intrusive thoughts, avoidance, and disturbances of identity. Addressing these transdiagnostic overlapping symptoms may therefore be key for treatment and intervention, particularly in younger populations where online peer support heavily shapes help-seeking behavior.

When considering negative associations, the Reddit network revealed specific points of disconnect between certain disorders and the broader network. Disorders related to substance use and behavioral addictions consistently appeared underconnected or negatively associated with other mental health conditions. This is in sharp contrast with comorbidity estimates typically reported in previous research, according to which substance use disorders often co-occur with other mental health conditions. For example, population-based estimates indicate that roughly 1 in 4 individuals with a substance use disorder have a comorbid mental disorder [66,67]. Our contrasting results might reflect a tendency of individuals in these communities to focus more narrowly on managing acute behavioral symptoms or crises rather than looking into their mental health more broadly. Their relative isolation may also stem from prevailing stigmatization and limited self-recognition or acknowledgment of co-occurring mental health issues, which together tend to position externalizing disorders outside the domain of conventional psychological problems [68-70]. Whether driven by a narrow focus on symptom management, social framing, or self-stigma, these isolating mechanisms risk reinforcing silos in both peer support and clinical care, obscuring potential links between addiction and other forms of psychopathology and making it more difficult to approach treatment holistically.

Beyond overall centrality, some disorders showed distinct patterns of connectivity, forming disproportionately strong links across the F code diagnostic categories while remaining relatively weakly integrated within their own. These bridging conditions included F43.1 (PTSD), F51.0 (nonorganic insomnia), and F33.8 (used to denote premenstrual dysphoric disorder here). They illustrate transdiagnostic mechanisms that current categorical frameworks do not explicitly capture, whether through hormonally linked mood dysregulation, sleep disturbances that cut across almost all clinical categories, or trauma‑related symptoms that span affective, anxiety, dissociative, and personality domains [71,72]. Notably, this pattern extended beyond individual conditions to entire diagnostic categories: all disorders in F2 (schizophrenia, schizotypal, and delusional disorders), F3 (mood and affective disorders), and the F60 subcategory (personality disorders) displayed more intercategory than intracategory links. Such patterns suggest particularly permeable diagnostic boundaries for these categories, a result that has also been observed at the level of diagnostic criteria [73].

The looseness of diagnostic boundaries was also observed through the comparative analysis between the Reddit network and the *ICD-10* network based on diagnostic criteria. The Reddit network displayed only slightly higher intercategory connectivity than the *ICD* diagnostic criteria network (68% vs 65% of all edges). However, the comparative hierarchical clustering analysis revealed that the Reddit network produced a substantially different hierarchy of disorders that only partially aligns with *ICD‑10* diagnostic structures. The 2 networks showed only low to moderate similarity in their clustering (ARI=0.295 and NMI=0.676), with only 13% of edges present in both networks. Reddit also exhibited lower modularity than *ICD-10*, meaning that these clusters were less separated by diagnostic category and more interconnected across them (normalized weighted modularity of 0.444 compared to 0.521). In addition, key divergences emerged at the level of disorders. Conditions such as F21 (schizotypal disorder), F84 (autism spectrum), and F90.0 (ADHD) were central and well‑integrated within the Reddit network but did not form cohesive clusters within the *ICD*‑based hierarchy of diagnostic criteria. Qualitative analyses of Reddit discussions similarly report tensions between lay and professional expertise [74], while quantitative comparisons indicate that certain conditions (such as anxiety-related and affective disorders) are disproportionately represented relative to registry data [75]. Such discrepancies may stem from the platform’s affordances of anonymity, its demographic composition, and its emphasis on peer support, but they may also signal blind spots in clinical frameworks. However, rather than contradicting established evidence, these divergences show how online data can surface experiential transdiagnostic mechanisms that remain underrepresented within formal diagnostic systems.

Despite clear differences between the Reddit network and the ICD-based network of diagnostic criteria, both point to the difficulty of fitting mental disorders into rigid, discrete categories [76,77]. While neither network should be treated as a ground truth of interdisorder relationships, the interconnected structure observed in both aligns with longstanding critiques that current psychiatric nosology underestimates the interconnected nature of psychopathology [78-81] and supports discussions of alternative paradigms, such as dimensional models that emphasize broad spectra and shared underlying features [82].

### Limitations and Future Directions

While our findings provide a large-scale view of the interconnected structure of psychopathology through mental health communities, several limitations should also be acknowledged. Our analysis is based on Reddit, a platform whose user base skews toward younger, Western, male, and digitally literate populations, which limits generalizability [83,84]. Reddit-specific dynamics such as anonymity, community norms, and moderation practices also shape what is shared and who participates. These features increase accessibility and ease of self-disclosure, but they also raise validity concerns, including account transience and the use of throwaway accounts that make authenticity difficult to assess [14,85]. Previous work also suggests that individuals with broader lay concepts of disorders are more likely to self-diagnose [86], which may amplify certain demographic biases. While these factors may contribute to divergences between the Reddit-derived and clinical structures, future work should clarify whether they reflect robust disorder associations or platform-specific outcomes. Hence, observed results should not be interpreted as verified comorbidities, but rather as behavioral signals that approximate perceived relatedness between disorders within digital contexts.

The methodological decisions delimit the scope of our results. We analyze posts, but not comments, made in 2022 within mental health support subreddits, and include only communities that map to a distinct disorder category in the *ICD-10*. Focusing on posts aligns with our aim to capture self-disclosure and help-seeking at the point of initiation, though it necessarily excludes peer interaction and information diffusion occurring in comment threads. Restricting to *ICD*-mapped subreddits increases construct clarity but may underrepresent transdiagnostic communities (such as r/MentalHealthSupport) or symptom-focused communities (such as r/SuicideWatch). Limiting the data to 2022 reduces temporal confounding from earlier structural instability, demographic shifts, and disruptions during the COVID-19 pandemic, and it ensures comparability across subreddits within a single, more mature phase of the platform. However, this temporal focus also constrains interpretation: network patterns and discourse are dynamic, and analyses spanning multiple years could reveal different structures as community composition and cultural context evolve. The year 2022 was chosen to provide a stable and interpretable baseline, but not to imply that the resulting associations are fixed over time.

Looking ahead, several promising directions emerge for future research. Reddit data offers a powerful proxy for examining mental health from multiple perspectives. Incorporating replies would add a complementary interaction layer, as comment threads reveal who engages with whom, what types of support are exchanged (eg, validation or advice), and how these interactions relate to subsequent posting trajectories. Temporal analyses could further enrich this view on 2 fronts. At the societal level, they would capture how mental health concepts and discourse evolve with cultural and technological change. At the individual level, following users over time could help distinguish between comorbidity, diagnostic progression, and broader help-seeking patterns, adding precision to how disorder associations are interpreted. Extending this approach beyond Reddit to platforms with different affordances and user bases would test how platform design shapes the organization of mental health discourse. We chose *ICD-10* as the reference framework for its international coverage and compatibility with registry data. However, comparable analyses using *DSM-5* (Diagnostic and Statistical Manual of Mental Disorders [Fifth Edition]), the forthcoming *ICD-11*, and other evolving diagnostic systems will be essential to assess psychiatry’s continuing efforts toward more coherent and empirically grounded concepts of mental health [11]. Such extensions could further reinforce the value of online mental health data as a bridge between peer discourse and clinical knowledge.

### Conclusions

This work maps a large-scale structure of mental health communities as they currently grow outside clinical settings, highlighting the necessity of perspectives that extend beyond formal diagnostic frameworks to achieve a more complete population-level understanding of psychopathology. Diagnostic frameworks remain essential, but they capture only part of how distress is articulated and managed in practice. Outside formal care, people navigate symptoms and negotiate meaning while seeking peer communities that mediate their mental health challenges. Digital platforms such as Reddit have become central to this process: they provide spaces for disclosure and support while also shaping the categories, language, and norms through which psychological distress is understood. In this sense, they are not only mirrors of cultural shifts but also infrastructures that reorganize how mental health is lived and discussed. Neglecting these platforms as legitimate sites of knowledge risks leaving research and practice poorly aligned with mental health needs amid rapid technological change.

## Appendix

Multimedia Appendix 1 (Top left) Distribution of the number of self-focused pronouns (I, me, myself) and other-focused pronouns used per post in the selected subreddits, capped at 20 occurrences. The density on the y-axis represents the relative proportion of posts at each pronoun count, rather than raw counts. Posts tend to include more self-focused pronouns (blue) compared to other-focused pronouns (red). (Top right) Overall proportion of posts showing different pronoun balance patterns: more self-focused pronouns (Self > Other), more other-focused pronouns (Other > Self), or balanced/none (Equal/Zero). The majority of posts are self-focused. (Bottom) Pronoun balance patterns stratified by ICD-10 diagnostic categories (Level 2). Each bar represents the proportion of posts within a diagnostic category that are more self-focused, more other-focused, or balanced. Across nearly all categories, self-focused pronouns dominate, but the relative proportions vary slightly between diagnostic groups.

Multimedia Appendix 2 Supplementary Tables (.xlsx): - ICD-10 Disorder Reference List - Mapping of Subreddits to ICD-10 and ICD-11 - List of (Weighted) Degrees (The Reddit Network of Psychopathology, Positive Associations) - List of Negative Edges and their Weights (Reddit Network of Psychopathology, Negative Associations). - ICD-10 Diagnostic Criteria.

Multimedia Appendix 3 Number of posts and subreddits across ICD-10 (International Classification of Diseases, 10th Revision) Chapter F mental health–related categories.

Multimedia Appendix 4 Online Interactive Tool - Map of Associations: Screenshot of the interactive Retina interface showing the node F32–F33 Depression (episodic and recurrent) selected as an example. The left-hand panel displays node-level metrics, including the number of users who posted in the corresponding subreddit(s), the weighted degree (sum of edge weights), the clustering coefficient (indicating local cohesiveness), and the number of triangles (three-node loops) formed around the node. This illustrates how the interface allows for exploratory analysis of individual disorder communities within the broader network structure.

Multimedia Appendix 5 Online Interactive Tool (Map of Disorder Associations): An interactive web-based visualization of the inferred disorder association network, allowing users to explore positive and negative associations, node connectivity, and diagnostic groupings derived from Reddit coposting activity.

Multimedia Appendix 6 No evidence of correlation between the number of users active in relation to a specific disorder (node size) and the number of links (node degree) in the Reddit network of psychopathology associations. The result supports the robustness of the inference method, suggesting that node centrality is not driven by subreddit size. Spearman correlation: r=0.068, p=0.64.

Multimedia Appendix 7

## Abbreviations

ADHD: attention-deficit/hyperactivity disorder
ARI: Adjusted Rand Index
DSM: Diagnostic and Statistical Manual of Mental Disorders
DSM-5: Diagnostic and Statistical Manual of Mental Disorders (Fifth Edition)
ICD: International Classification of Diseases
ICD-10: International Statistical Classification of Diseases, 10th Revision
ICD-11: International Classification of Diseases, 11th Revision
NMI: normalized mutual information
PTSD: posttraumatic stress disorder
TCPS 2: Tri-Council Policy Statement: Ethical Conduct for Research Involving Humans

## Data Availability

Subreddit metadata and aggregated data supporting the findings of this study are provided in the corresponding Multimedia Appendices. Due to the platform’s terms of service, raw Reddit post content cannot be publicly shared. Additional aggregated data may be made available upon reasonable request by contacting the corresponding author.
