# Supplementary figures and images for: The Structure of Psychopathology on Reddit: Network Analysis of Mental Health Communities in Relation to the ICD Diagnostic System
Source: J Med Internet Res. 2026 Jan 30;28:e80958. doi: 10.2196/80958 (PMC12905569; doi:10.2196/80958)

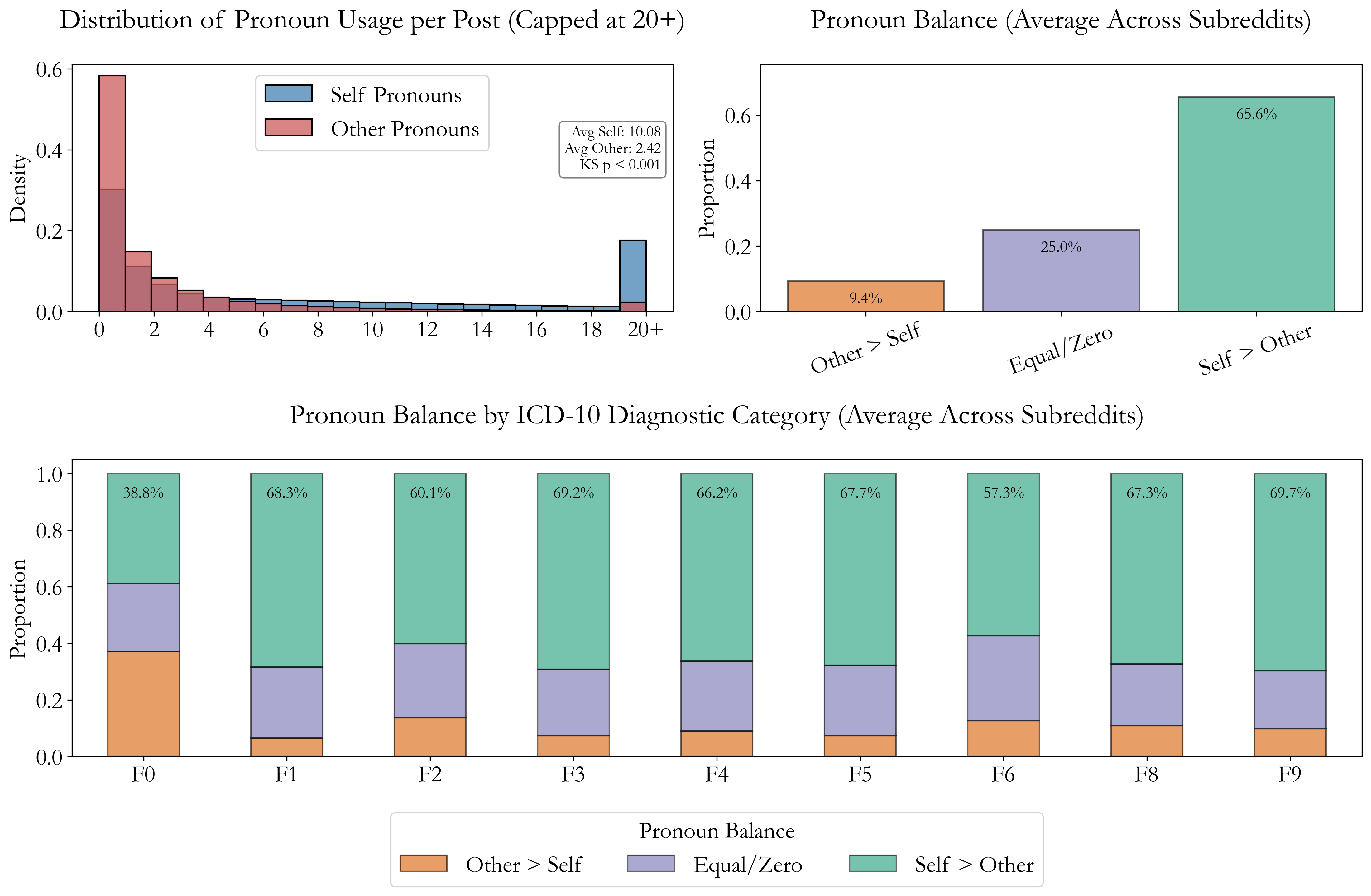

Supplement: Multimedia Appendix 1 [file jmir_v28i1e80958_app1.png]

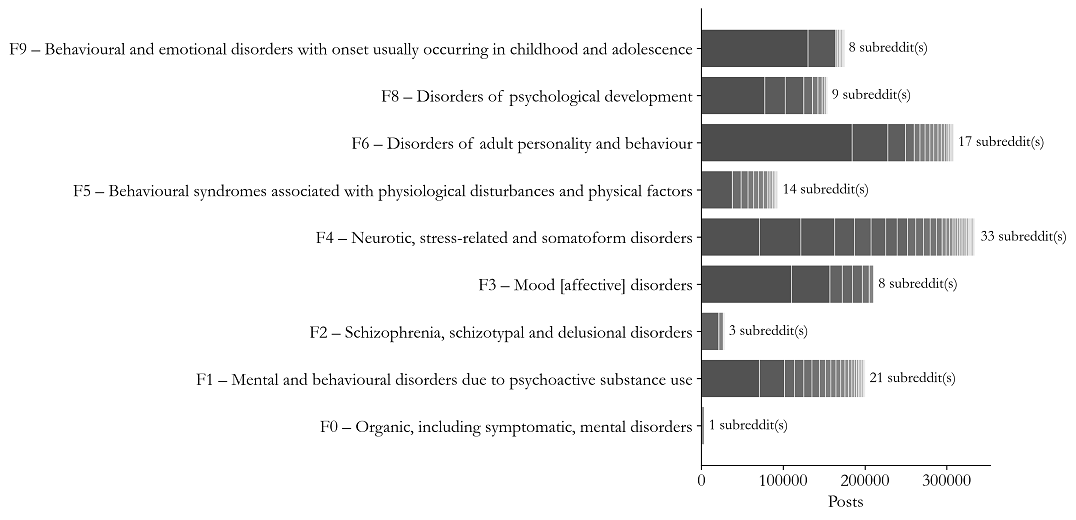

Supplement: Multimedia Appendix 3 [file jmir_v28i1e80958_app3.png]

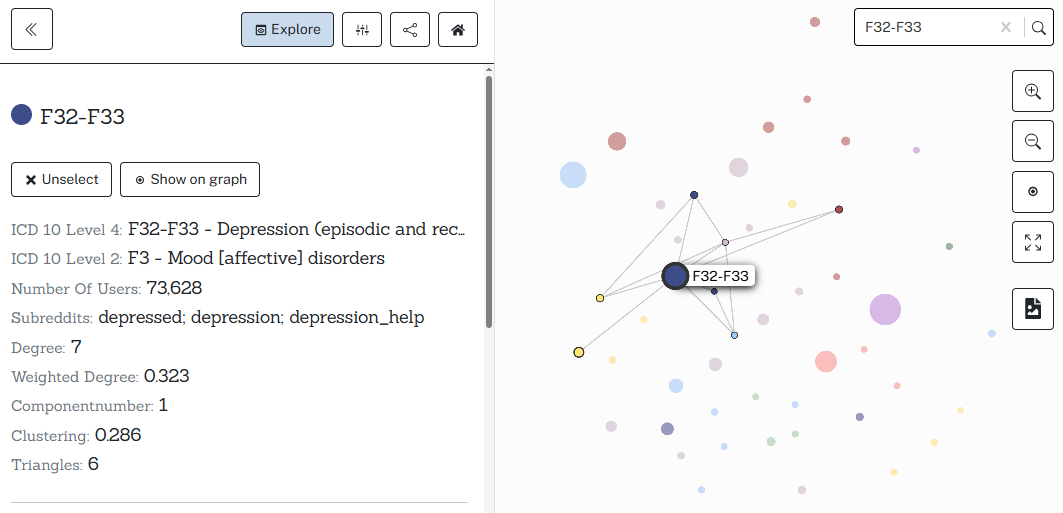

Supplement: Multimedia Appendix 4 [file jmir_v28i1e80958_app4.png]

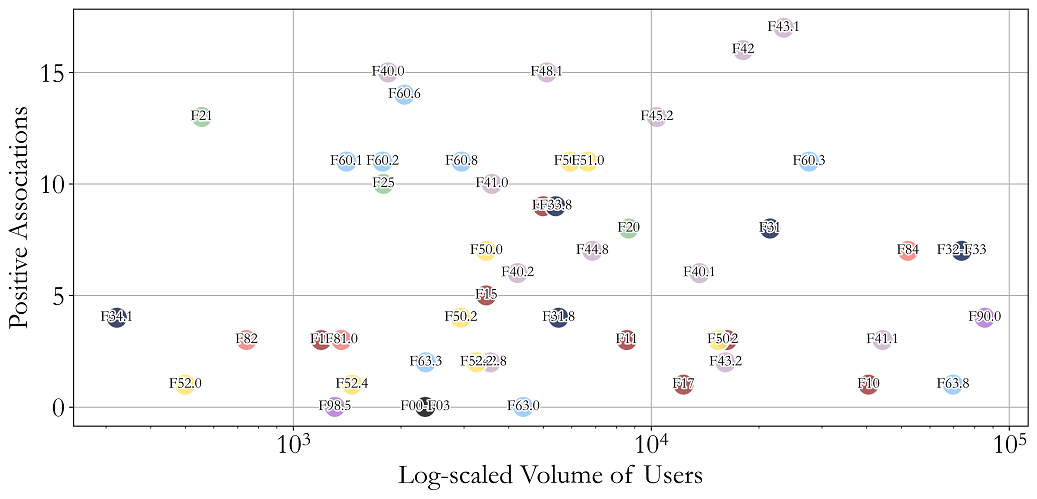

Supplement: Multimedia Appendix 6 [file jmir_v28i1e80958_app6.png]

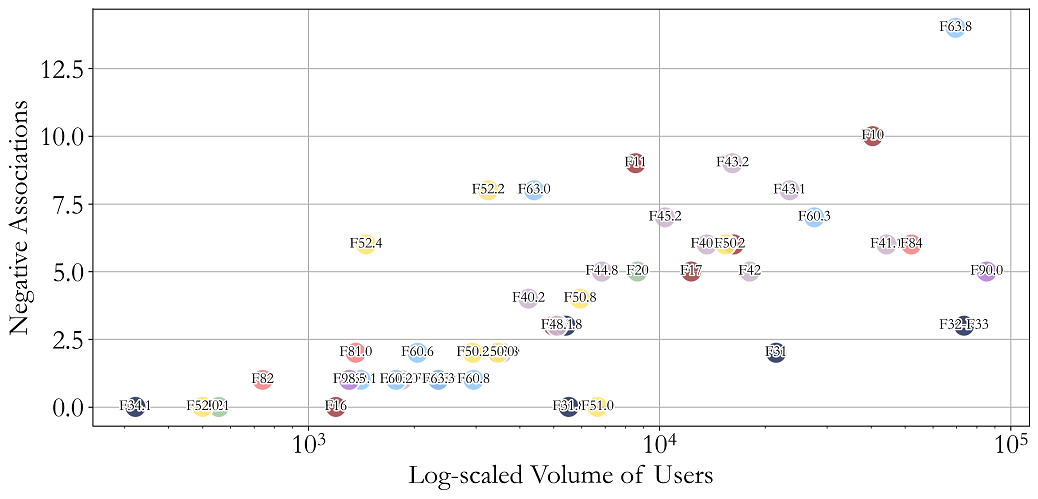

Supplement: Multimedia Appendix 7 [file jmir_v28i1e80958_app7.png]
