# Supplementary material for: The Structure of Psychopathology on Reddit: Network Analysis of Mental Health Communities in Relation to the ICD Diagnostic System
Source: J Med Internet Res. 2026 Jan 30;28:e80958. doi: 10.2196/80958 (PMC12905569; doi:10.2196/80958)
Supplement: Multimedia Appendix 5 [file jmir_v28i1e80958_app5.docx]

## Supplementary Materials: Online Interactive Tool - Map of Associations

Interactive versions of the three main networks analyzed in this study are available online:

- **Reddit Network of Psychopathology (Positive Associations)**
  Displays statistically significant co-posting links between disorder-specific communities.
  [Interactive](https://ouestware.gitlab.io/retina/beta/#/graph/?url=https%3A%2F%2Fgist.githubusercontent.com%2Fboevkoski%2F2903ebeb33e7a5462cd6597c50579033%2Fraw%2Ff2193e5b53434fe33751cd2df80ccf3cbd78d889%2Freddit_psychopathology_positive_associations.gexf) | [Gist](https://gist.github.com/boevkoski/2903ebeb33e7a5462cd6597c50579033)
- **Reddit Network of Psychopathology – Negative Associations**
  Shows significant under-representation of shared users between disorder communities.
  [Interactive](https://ouestware.gitlab.io/retina/beta/#/graph/?url=https%3A%2F%2Fgist.githubusercontent.com%2Fboevkoski%2F4b35e1816e07076a1aa80ad03a4e1a2b%2Fraw%2F2d122d931cfe6944c4d9c51713d77357a6d44e47%2Freddit_psychopathology_negative_associations.gexf) | [Gist](https://gist.github.com/boevkoski/4b35e1816e07076a1aa80ad03a4e1a2b)
- **Network Based on Diagnostic Criteria (ICD-10)**
  Visualizes the co-diagnosis network derived from symptom-based similarity of disorders.
  [Interactive](https://ouestware.gitlab.io/retina/beta/#/graph/?url=https://gist.githubusercontent.com/boevkoski/4423ac95663e168e2d355b12d96a7a6f/raw/feee54a83186335facaf89c5133f6673a95ed6b8/network-cefbbb22-adc.gexf) | [Gist](https://gist.github.com/boevkoski/4423ac95663e168e2d355b12d96a7a6f)

Each network can be explored interactively through Retina, where node size indicates subreddit volume or diagnostic relevance, edge weight reflects association strength, and color encodes ICD-10 categories.


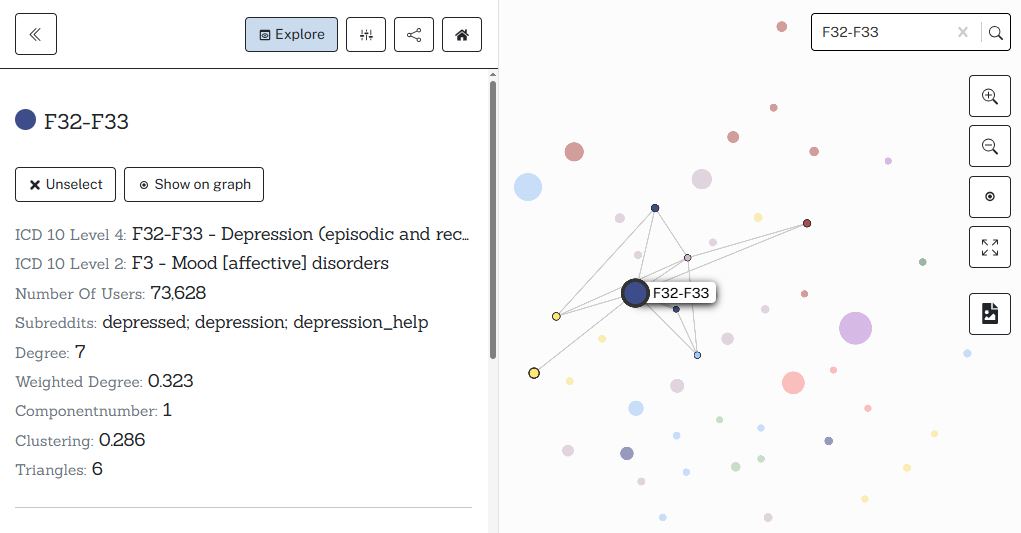


Supplementary Figure 5: Screenshot of the interactive Retina interface showing the node F32–F33 Depression (episodic and recurrent) selected as an example. The left-hand panel displays node-level metrics, including the number of users who posted in the corresponding subreddit(s), the weighted degree (sum of edge weights), the clustering coefficient (indicating local cohesiveness), and the number of triangles (three-node loops) formed around the node. This illustrates how the interface allows for exploratory analysis of individual disorder communities within the broader network structure.
